# Current RT-qPCR to detect SARS-CoV-2 may give positive results for related coronaviruses

**DOI:** 10.1007/s00203-022-03029-y

**Published:** 2022-06-23

**Authors:** Antonio Martínez-Murcia, Adrián García-Sirera, Aaron Navarro, Laura Pérez

**Affiliations:** 1 Department of Microbiology, University Miguel Hernández, 03312 Orihuela, Alicante, Spain; 2 Genetic PCR Solutions™, 03206 Elche, Alicante, Spain

**Keywords:** Reverse transcription qPCR, SARS-CoV-2, Coronavirus, Specificity, False positives

## Abstract

Some weeks after the first CoVID-19 outbreak, the World Health Organization published some real-time PCR (qPCR) protocols developed by different health reference centers. These qPCR designs are being used worldwide to detect SARS-CoV-2 in the population, to monitor the prevalence of the virus during the pandemic. Moreover, some of these protocols to detect SARS-CoV-2 have widely been applied to environmental samples for epidemiological surveillance purposes. In the present work, the specificity of these currently used RT-qPCR designs was validated in vitro using SARS-CoV-2 and highly related coronaviral genomic sequences and compared to performance of the commercially available GPS™ CoVID-19 dtec-RT-qPCR Test. Assays performed with SARS-CoV-2-related genomes showed positive amplification when using some of these qPCR methods, indicating they may give SARS-CoV-2 false positives. This finding may be particularly relevant for SARS-CoV-2 monitoring of environmental samples, where an unknown pool of phylogenetically close-related viruses may exist.

## Introduction

The pandemic Coronavirus Disease 2019 (CoVID-19), caused by the Severe Acute Respiratory Syndrome Coronavirus 2 (SARS-CoV-2), has led to more than 364 million confirmed cases and more than 5.6 million deaths as of 28th January 2022 (http://covid19.who.int). SARS-CoV-2 belongs to the *Betacoronavirus* genus and is a member of the Severe Acute Respiratory Syndrome-related coronavirus species which includes also SARS-CoV and several strains of similar viruses isolated from bats and pangolins (Ceraolo and Giorgi 2020; Zhou et al. 2020; World Health Organization 2020a).

At the beginning of the first outbreak, with the publication of the first SARS-CoV-2 genome (GenBank accession no: MN908947), reverse transcription qPCR (RT-qPCR) and end-point PCR protocols to detect SARS-CoV-2 (World Health Organization 2020b) were developed by reference laboratories such as Charité Virology, Berlin, Germany (targets E and RdRp with P1 and P2 probes); Institut Pasteur, Paris, France (IP2 and IP4); Centers for Disease Control and Prevention (CDC), Division of Viral Diseases, Atlanta, USA (N1 and N2); China (ORF1ab and N); Hong Kong University (ORF1b and N); Department of Medical Sciences, Ministry of Public Health, Thailand (N); the National Institute of Infectious Diseases, Japan (N) (World Health Organization 2021). Consequently, these RT-qPCR designs set the grounds for many PCR-based commercial kits and detection methods, enabling global testing of SARS-CoV-2 in clinical and environmental samples, such as urban wastewater samples.

Controlling the spread of the virus was challenged by a lack of an easy symptom-based diagnosis (particularly at early stages of the infection), a considerable asymptomatic population, and the widespread nature of the pandemics. Regardless of that RT-qPCR tests are useful tools to control the spread of isolated clinical cases, when analyzing environmental samples, the exclusivity of the primers could be affected by the presence of undescribed microbial diversity. Altogether, this has led to the enforcement of radical and extremely costly epidemiological control measures, including contact tracing, lockdowns, and curfews. Therefore, setting up workable and reliable methods for SARS-CoV-2 epidemiological surveillance is necessary to control and prevent new outbreaks through the appliance of preventive measures.

Various studies reported that both asymptomatic and recently recovered patients can still excrete SARS-CoV-2 in feces and urine (Cheung et al. 2020; Lescure et al. 2020; Lo et al. 2020; Randazzo et al. 2020; Sun et al. 2020; Wang et al. 2020). Additionally, transmission of SARS-CoV-2, either directly through respiratory droplets or indirectly through fomites, is generally accepted because of the positive detection in samples from various environmental surfaces, air and sewage in hospital and community settings (Mouchtouri et al. 2020; World Health Organization 2020a). In line with this, it has generally been considered that environmental risk assessment could act as an effective epidemiological surveillance tool (Andrews et al. 2020). However, when testing SARS-CoV-2 by RT-qPCR in environmental samples, the exclusivity of primers/probe sequences becomes extremely relevant due to the huge viral diversity that exists in nature, compared to clinical samples. In a previous paper, we analyzed in silico the exclusivity of some RT-qPCR designs widely used to detect SARS-CoV-2 RNA published by WHO, compared to the GPS™ *CoVID-19 dtec-RT-qPCR Test* (Martínez-Murcia et al., 2020). Concerning the GPS™ kit, the design is periodically evaluated under a Post Market Surveillance Plan showing the assay is inclusive for all SARS-CoV-2 variants up to date.

In the present study, we aimed to analyze the exclusivity of the primers/probes released by WHO and this from the GPS™ CoVID-19 dtec-RT-qPCR against related SARS-CoV-2 coronavirus sequences as templates for RT-qPCR. Because, at the beginning of present study, the closest sequences to SARS-CoV-2 deposited in public databases were these of Pangolin CoV, DNA-synthetic constructs based on those sequences were built to achieve experimental tasks for assessing the present purpose.

## Materials and methods

### *In silico* analysis: blast search

Fragments of the SARS-CoV-2 sequence (NC_045512.2) corresponding to each PCR amplicon of the evaluated primers/probe designs (Centers for Disease Control and Prevention 2020; Corman et al. 2020; Institut Pasteur 2020; Martínez-Murcia et al., 2020) were analyzed independently to find highly similar sequences not belonging to SARS-CoV-2. Sequence search was made using the Basic Local Alignment Search Tool (BLAST) software available on the National Center for Biotechnology Information (NCBI, https://blast.ncbi.nlm.nih.gov/Blast.cgi) website databases (Bethesda, MD, USA) selecting the Nucleotide Collection database and optimized with the Somewhat Similar Sequences algorithm (blastn). SARS-CoV-2 sequences were excluded from the analysis to obtain the most similar non-SARS-CoV-2 sequences from the database. The closest non-SARS-CoV-2 sequence was selected and used to test their in vitro specificity.

### In vitro analysis

#### Pangolin CoV synthetic DNA templates

To test the exclusivity of the RT-qPCR designs in vitro, synthetic DNA templates were provided by IDT (California, USA) and Eurofins Genomics Germany GmbH (Ebersberg, Germany). Two templates were synthesized to test each RT-qPCR design, one containing a full match sequence (positive control) and another corresponding to the most closely related sequence of the selected non-SARS-CoV-2 strain. A non-related quality control sequence belonging to *Salmonella* genus was added to all synthetic templates to calibrate the template copy number with another set of primers.

#### Primers and probes for RT-qPCR

Primers and probes of IP2 and IP4 designs were provided by IDT (California, USA), and these of RdRp-P2, N1, and N2 were obtained from Eurofins Genomics Germany GmbH (Ebersberg, Germany). Finally, *GPS™ CoVID-19 dtec-RT-qPCR Test* was supplied by Genetic Analysis Strategies S.L. (GPS™; Alicante, Spain).

#### RT-qPCR protocol

DNA-synthetic templates were diluted with DNase/RNase free water by preparing tenfold dilution series to finally obtain *ca.* 10–10^6^ total copies on the qPCR reactions. As recommended for each RT-qPCR design, we used an amplification step of 50 cycles at 95ºC for 15 s and at 58ºC for 30 s for IP2 and IP4 (Institut Pasteur 2020); 45 cycles at 94ºC for 15 s and at 58ºC for 30 s for RdRp-P2 (Corman et al. 2020); and 45 cycles at 95ºC for 3 s and at 55ºC for 30 s for N1 and N2 (Centers for Disease Control and Prevention, 2020). Finally, the GPS™ *CoVID-19 dtec-RT-qPCR Test* was subjected to 45 cycles at 95ºC for 10 s and at 60ºC for 60 s following the manufacturer’s instructions. Both, reverse transcription, and activation steps were performed according to the LyoMix RT-qPCR master mix (GPS™; Alicante, Spain), used in all RT-qPCR tests, at 50ºC for 10 min and 95ºC for 1 min. Positive and negative PCR controls were included, and reaction mixtures were subjected to qPCR in a StepOne thermocycler (Applied Biosystems, USA).

## Results

### qPCR designs alignment with pangolin CoV

The alignments were performed using the MEGA 5.2.2 software (Tamura et al. 2011) with the GPS™ RT-qPCR target sequence and each target-sequence region comprising the primers and probes published by WHO. The alignments showed that Pangolin CoV isolate MP789 (GenBank accession No.: MT121216.1) contained the most similar target sequence for IP2, IP4,N1, E and RdRp-P2 RT-qPCR designs; Pangolin CoV isolate PCoV_GX-P2V (GenBank accession No.: MT072864.1) the most similar target for N2; and hCoV-19/pangolin/Guangxi/P1E/2017 (GISAID Accession ID: EPI_ISL_410539) for GPS™ *CoVID-19 dtec-RT-qPCR Test*. These three sequences were selected to synthesize the corresponding DNA templates.

### *In silico* analysis of primers and probes

**Fig. 1:**
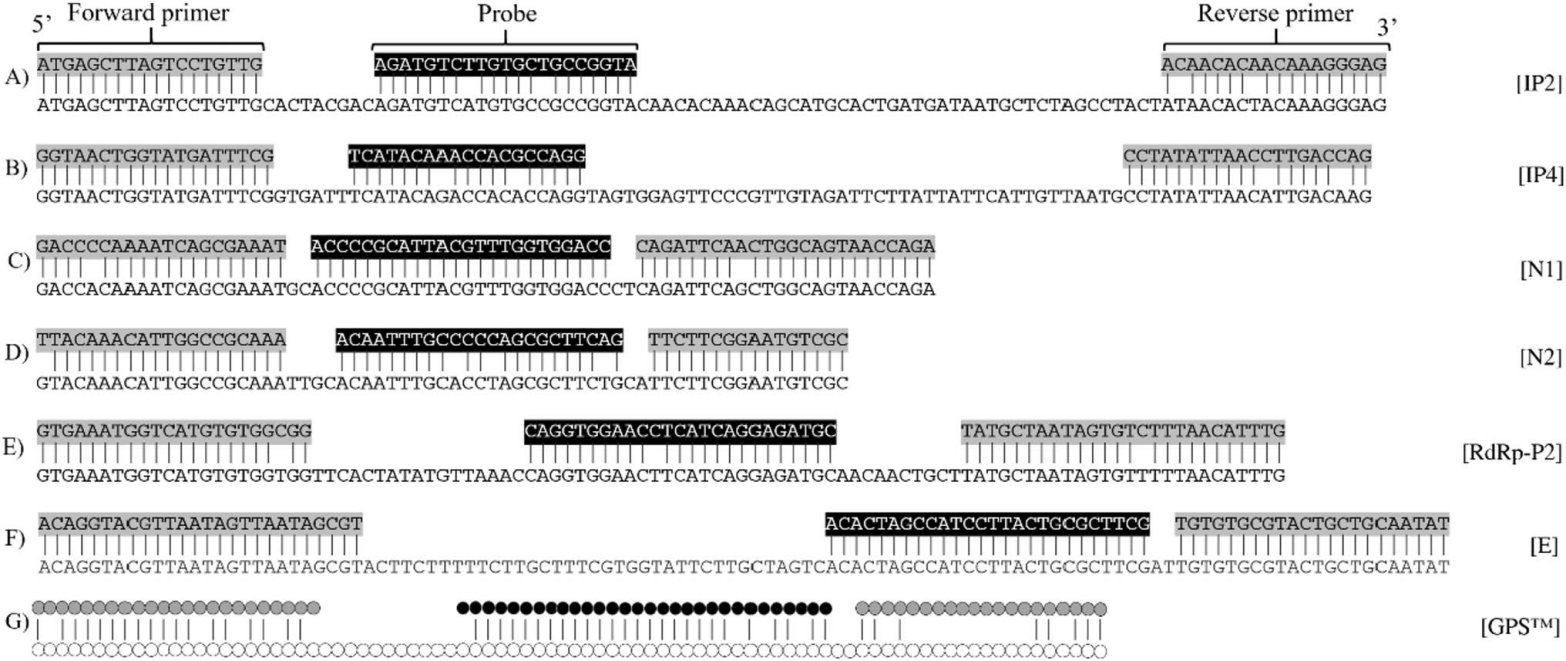
Sequence alignment between primers and probes: **a**) IP2 with Pangolin CoV—MT121216.1; **b**) IP4 with Pangolin CoV—MT121216.1; **c**) N1 with Pangolin CoV—MT121216.1; **d**) N2 with Pangolin CoV—MT072864.1; **e**) RdRp-P2 with Pangolin CoV—MT121216.1; **f**) E with pangolin CoV—MT121216.1; **g**) GPS™ *CoVID-19 dtec-RT-qPCR Test* with Pangolin CoV—EPI_ISL_410539

The sequence alignments of primers/probe with the corresponding selected Pangolin CoV sequence (Fig. 1) indicated that both IP2 and IP4 qPCR designs showed 4 mismatches, 2 located in probe and 2 in reverse primer; N1 showed 2 mismatches, 1 in each primer; N2 showed 4 mismatches, 1 in forward primer and 3 in probe; RdRp-P2 showed 3 mismatches, 1 in each primer and 1 in probe; E showed full match; finally, GPS™ *CoVID-19 dtec-RT-qPCR Test* showed a total of 21 mismatches, mostly located in the reverse primer. As the E design showed full matching with the selected coronavirus sequences, its corresponding in vitro test was excluded from this analysis.

### In vitro* analysis*

**Fig. 2:**
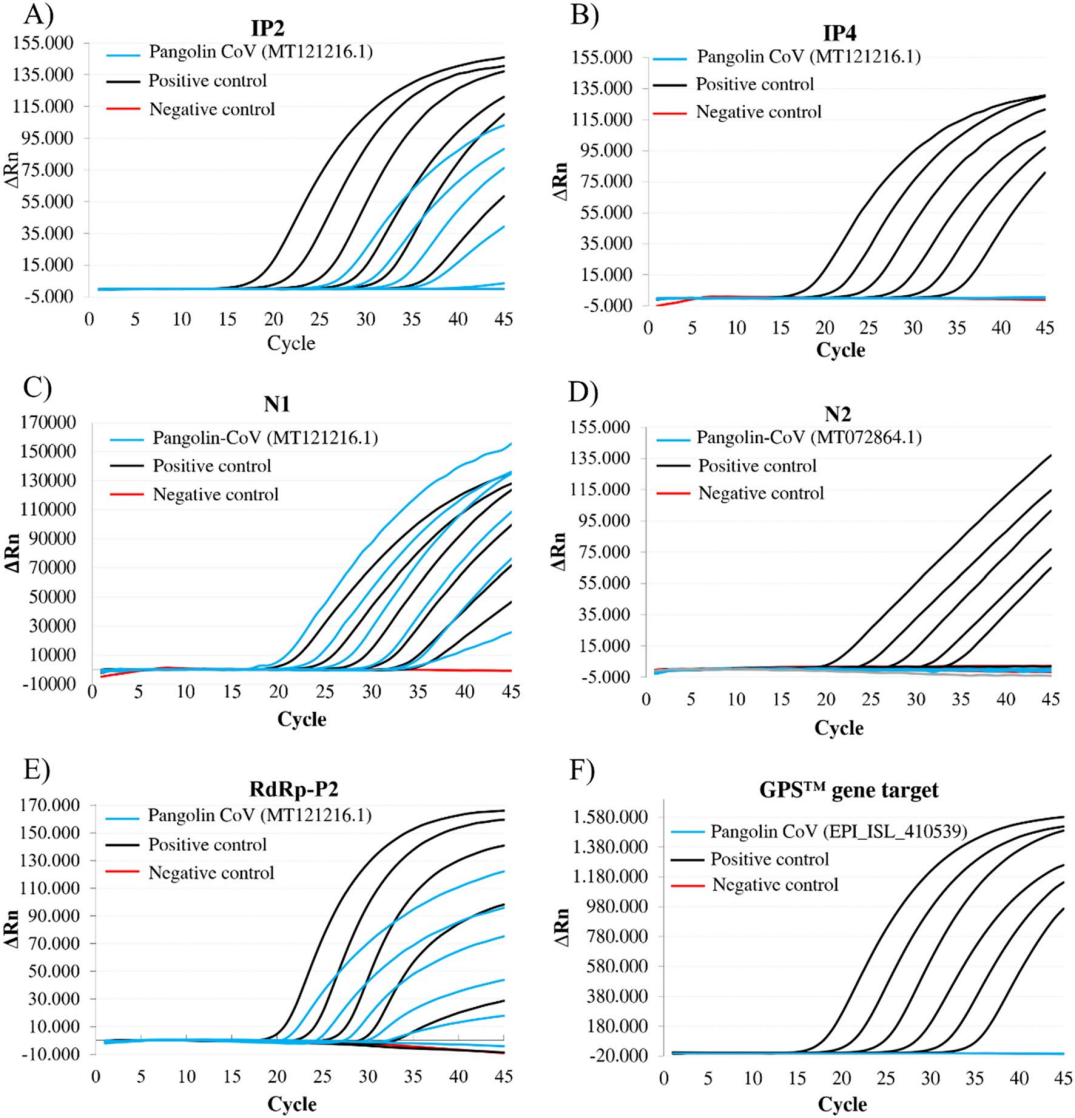
Amplification plots of synthetic DNA template dilutions from 10 to 10.^6^ copies of positive controls and Pangolin CoV nucleotide sequences, and negative controls, using the **a**) IP2; **b**) IP4; **c**) N1; **d**) N2; **e**) RdRp-P2; **f**) GPS™ *CoVID-19 dtec-RT-qPCR Test* qPCR designs

The in vitro exclusivity analysis of all RT-qPCR designs under study was carried out by testing the specificity of each primer/probe using 10–10^6^ copies of the synthetic DNA as template (Fig. 2). Positive controls for all RT-qPCR tests were performed using synthetic templates with sequences showing full match to the corresponding RT-qPCR design. N1 and RdRp-P2 designs (with 2 and 3 mismatches, respectively) resulted in Ct values close to those of the corresponding positive controls (Ct ± 1) which suggested a relatively stable hybridization of the oligonucleotide primers and probes with the target sequences. However, the IP2 RT-qPCR design (showing 4 mismatches) yielded higher Ct values compared to the positive control. IP4 and N2, (both showing 4 mismatches) showed no qPCR amplification. Additionally, an end-point PCR was performed with primers of IP4 and N2 (excluding the corresponding probes) and very robust bands of the expected fragment size were obtained in the agarose gels (data not shown). The GPS™ *CoVID-19 dtec-RT-qPCR Test* (21 mismatches) showed no amplification. All the experiments were repeated in three independent runs, by different technicians on different days and results were reproducible in all cases.

## Discussion and conclusions

On 14th January 2020, the WHO published a variety of RT-qPCR and end-point PCR protocols to diagnose COVID-19 World Health Organization, 2020b). In the present study, we have analyzed the exclusiveness for SARS-CoV-2 detection of three RT-qPCR designs: Institut Pasteur (Paris), CDC from Atlanta and Charité (Berlin), and this from a commercially available kit (GPS™ CoVID-19 dtec-RT-qPCR Test, Alicante, Spain). In silico analyses were performed to identify non-SARS-CoV-2 coronaviruses with the highest percentage of homology at the targets used to identify SARS-CoV-2. Three CoV sequences from Pangolin (isolates MP789, PCoV_GX-P2V, and hCoV-19/pangolin/Guangxi/P1E/2017) and this of bat CoV RaTG13 were further analyzed to evaluate the specificity of the RT-qPCR designs in vitro. Despite that at the time of the analysis, the bat RaTG13 sequence showed the highest homology (96.2%) to SARS-CoV-2, it was not selected to be used in our experiments. The sequence was excluded because only a single RaTG13-like sequence (Zhou et al., 2020), showing this level of similarity has been described so far, and the possibility of contamination during genome sequencing should not be ruled out. Moreover, although determined from a sample taken in July 2013, the bat RaTG13 sequence was first reported on January 2020 (MN996532.1). A posterior modification in October 2020 (MN996532.2) on the NCBI database showed nucleotide differences at six positions which, to some extent, reaffirm our preliminary concerns. Finally, RaTG13 sequence was excluded in the present study as it showed full matching to IP2, N2, E, RdRp-P2, and GPS™ RT-qPCR targets.

Detection on nature (environmental samples) includes full genomes of the target, and highly similar, and other variety of organisms as well as complex matrix. Samples should be properly processed and purified to achieve detection by qPCR. Despite all these complexities, the method was simplified using pure DNA fragments, rather than RNA, to assess the cross reactivity between the pangolin CoV segments and the primers. Nevertheless, despite using a DNA template, the RT step was maintained as it was part of the master mix protocol used to run all PCRs.

Results obtained from the RT-qPCR tests indicated that IP2, N1 and RdRp-P2 RT-qPCR designs were able to amplify, at reference PCR conditions, the selected Pangolin CoV sequences. This fact reinforces the hypothesis of possible false positives when testing exclusively for SARS-CoV-2 in environmental samples. The IP4 and N2 RT-qPCR designs (Fig. 2) yielded specific results when evaluated, but their specificity was further evaluated with an end-point PCR, obtaining positive bands of the expected size (data not showed). These findings suggested that amplification may occur in the qPCR, but the discriminative character may reside on the probe (2 and 3 mismatches, respectively). We speculate that the IP4 assay works on restrictive conditions (58.0ºC) due to the low Tm of the IP4 probe (56.0ºC), which could decrease its affinity to the template when at least two mismatches are present. Regarding the N2 probe, two mismatches are located on a GC enriched position, another mismatch close to the 3’-end of the oligo which could be preventing the probe to attach efficiently to the template, thus resulting in a lack of signal. The GPS™ *CoVID-19 dtec-RT-qPCR Test* showed no amplification when tested against the most similar described sequences.

Due to the low number of mismatches found in the alignments between primers/probes and the Pangolin CoV sequences, we considered the possibility that IP2, IP4, N1, and RdRp-P2 may amplify from samples containing some Pangolin-like CoVs, or other non-described viral strains with similar sequences (Fig. 1). Altogether, the in vitro results showed that some RT-qPCR designs may be able to detect Pangolin-like CoV sequences, or highly related, giving rise to false positives when testing SARS-CoV-2 by RT-qPCR in environmental samples.

Even though these findings may have low relevance on clinical diagnostics, studies in mink farms suggested that transmission of animal-specific coronaviruses cannot be disregarded (Munnink et al. 2021). Therefore, the exclusivity of the RT-qPCR design is relevant for SARS-CoV-2 monitoring in environmental samples, particularly in the epidemiological surveillance of wastewaters. Several laboratories have developed protocols for water sample analysis using some of the analyzed RT-qPCR designs to detect SARS-CoV-2 (Chavarria-Miró et al. 2020; Fongaro et al. 2020; Randazzo et al. 2020) evaluated in the present study. These results suggested the presence of SARS-CoV-2 in wastewater samples harvested in countries remotely distant from China (12th March 2019 and 27th November 2019), prior to the first outbreak was formally detected (31st December 2019). However, those results were supported by considerably low RT-qPCR signals, with Ct values ranging from 39 to 40 (Chavarria-Miró et al. 2020). Having ruled out a possible cross contamination during the PCR reactions, some of these weak positives could be the consequence of a lack of exclusivity. Although virus sequences from Pangolin should not be expected outside Asia and Africa, at least hypothetically, the presence of highly related sequences from undescribed coronavirus can be present in environmental samples.

The true extent of microbial diversity is still largely unknown as the vast extent of it is still to be described (Locey and Lennon 2016); therefore, it is crucial to consider that environmental samples may contain a pool of many different but closely related viruses. Consequently, a considerable number of different sequences were unknown when the RT-qPCR designs were developed. Moreover, in the span of a few months, since we started this study till December 2021, several non-SARS-CoV-2 betacoronavirus sequences have been deposited in public databases that have even higher levels of similarity towards the evaluated designs than these of the Pangolin CoV sequences evaluated in the present report. Some examples of sequences are the following bat coronavirus sequences (GenBank accession No.: MW201981.1, MZ081381.1, or MW251311.1).

While some of these RT-qPCR designs recommended by WHO showed a relatively low SARS-CoV-*2* exclusivity, the RT-qPCR test worldwide available by GPS™ was the most exclusive by far. These data highlight the relevance of using highly exclusive primers and probes to detect specifically the SARS-CoV-2 for pandemic surveillance. Finally, the present work emphasizes that studies, either aiming to discriminate between SARS-CoV-2 strains or evaluating the real diversity from environmental samples, should be complemented using massive sequencing techniques.

## Declarations

### Data availability

Most of the data that support the findings of this study are openly available in the NCBI repository at https://www.ncbi.nlm.nih.gov/: SARS-CoV-2 reference genome (NC_045512.2) GenBank accession no: MN908947: Pangolin CoV isolate MP789 (GenBank accession no.: MT121216.1). Pangolin CoV isolate PCoV_GX-P2V (GenBank accession no.: MT072864.1); and hCoV-19/pangolin/Guangxi/P1E/2017 (GISAID Accession ID: EPI_ISL_410539). WHO primers are publicly available (World Health Organization, 2020b). GPS™ CoVID-19 dtec-RT-qPCR Test primer sequences are not publicly available due to privacy restrictions but available from the corresponding author upon reasonable request.
